# Analysis of Factors Affecting the Preparation of Mullite Whiskers from Silica-Rich Slag and Application Studies

**DOI:** 10.3390/ma16247633

**Published:** 2023-12-13

**Authors:** Shangwen Zhu, Xiaohua Gu, Siwen Liu, Yan Liu

**Affiliations:** 1 School of Energy and Building Environment, Guilin University of Aerospace Technology, Guilin 541004, China; glzsw2337899@163.com (S.Z.); 2019009@guat.edu.cn (Y.L.); 2 State Key Laboratory for Modification of Chemical Fibers and Polymer Materials, College of Materials Science and Engineering, Donghua University, Shanghai 200051, China; 3 College of Innovative Material & Energy, Hubei University, Wuhan 430062, China; 202121113012770@stu.hubu.edu.cn

**Keywords:** mullite whiskers, molten salt system, ceramics, toughened, silica-rich slag

## Abstract

This paper presents an in-depth comparative study of the effects of different molten salt systems, catalyst additions, preparation temperatures, temperature rise rates, and holding times on the properties of mullite whiskers during their preparation process, as well as exploring the enhancement of the toughening effect of mullite whiskers on ceramics. The morphology, crystal structure, and composition of the whiskers were analyzed via SEM, XRD, TG, strength tests, etc. The results show that the best-performing mullite whisker was prepared with an aluminum sulfate molten salt system, with the addition of aluminum fluoride catalyst at 4%, a temperature increase rate of 5 °C, a temperature increase up to 850 °C, and a holding time of 5 h, and its aspect ratio reached 20.64. By adding different contents of mullite whiskers and comparing the toughness strengths and wear rates of the silicon carbide ceramics, it was found that the toughness strength of the ceramics was improved by more than 16.5% and the wear rate was lower than 0.4% when the addition of mullite whisker was more than 3%.

## 1. Introduction

The rapid development of the petrochemical field in recent years has resulted in an increasing demand for high efficiency and few by-products [1]. Catalysts [2,3] can satisfy these demands and improve energy consumption by virtue of the advantages of lower quantities being required, non-participation in the reaction itself, and changing the reaction rate. However, the production and use of catalysts also generates some waste [4,5], which usually contains catalyst components and other hazardous substances. Therefore, how to treat and reuse catalyst wastes has become an urgent problem.

Silica-rich waste gum sludge containing aluminum elements is generated during catalyst production and use [6]. Currently, the most commonly used treatment methods are still landfill and combustion. The landfill method [7,8] is contradictory to the original purpose of the treatment, as it leads to the pollution of soil and water resources. Chemical treatment [9,10] can generate high-value recycled products through chemical reactions based on the material composition of the waste, which is more in line with the concept of green and sustainable development. Mullite whiskers [11,12] are widely used as high-end toughened base materials [13,14] in aerospace, military, and chip applications by virtue of their high flexural and tensile mechanical properties, low thermal conductivity, high creep resistance, and low coefficient of thermal expansion. Also, the product is used in the surface coating [15] of equipment to increase its service life. Aluminum-containing silica-rich waste slag is mainly composed of alumina, silica, and a very small portion of metal oxides. Its chemical composition is rich in silicon and aluminum elements and has great potential as a silicon and aluminum source for mullite whisker preparation. However, the development of mullite whiskers is limited by the high-temperature preparation environment [16] and the cost of raw materials [17], which prevent large-scale production and toughening applications. Therefore, it is of great practical importance to prepare mullite whiskers with high reaction efficiency, low cost, and low temperature.

To date, mullite whisker preparation methods have mainly comprised mineral decomposition [18,19,20], the molten salt method [21], powder calcination [22], the solid–gel method [23,24], rare earth oxide doping [25,26,27], the co-precipitation method [28], and catalytic crystallization [29,30]. The most commonly used method is the mineral decomposition method because it uses a wide range of naturally occurring minerals, such as kaolin [31,32], bluestone [33], and hydrotalcite [34]. A number of scholars [35,36] have also recycled boiler plant waste through pulverized coal slag. Although the mineral decomposition method has become mainstream, given its advantages of low raw material costs and wide sources, the preparation of mullite whiskers is characterized by high production cost, high energy consumption for production, and non-uniform crystal morphology, and the development of new methods to prepare mullite whiskers with strong crystals and uniform distribution at low temperatures is still a great challenge. The molten salt method [37,38] promotes whisker growth due to its unique reaction atmosphere, but the reaction temperature is above 1200 °C. Many scholars have devoted themselves to the preparation of mullite whiskers using the low-temperature [39,40] molten salt method. Rashad et al. [41] prepared mullite whiskers by utilizing the catalytic effects of AlF_3_ in a reaction environment at 1100 °C. Wang et al. [42] grew mullite whiskers using Al_2_(SO_4_)_3_-Na_2_SO_4_ as a molten salt system at 1023 °C. Chen et al. [43] and Abdullayev et al. [44] further reduced the preparation temperature to below 900 °C by adding aluminum fluoride. At the same time, there are also many scholars [45] focusing on the influencing factors of mullite whisker growth, but they do not take into account the most important factors of the molten salt system, the rate of temperature rise, or the heating time as regards their effects on the structure of mullite, and studies have yet to be undertaken on the effects of mullite whiskers on ceramic toughness.

There are few reports on the preparation of mullite whiskers from different molten salt systems and their toughening optimization. Based on previous work on the low-temperature preparation of mullite whiskers from silica-rich and aluminum-containing colloidal slag, this paper further discusses the effects of the molten salt system, the temperature rise rate, the heating time, the catalyst content, and the reaction temperature on the growth of mullite whiskers. The mechanism of toughening ceramics with mullite whiskers is also investigated, and different whisker addition schemes are discussed. Research on the preparation of mullite and the optimization of its ceramic-toughening effects by different molten salt systems of silica-rich slag is of great strategic significance for the sustainable development of fields such as aerospace, the military industry, and chip manufacturing.

## 2. Experimental Section

This chapter focuses on the drugs, apparatus, preparation process, and reaction mechanism involved in the experiment.

### 2.1. Raw Materials and Reagents

The raw materials and reagents used in this experiment were as follows:

- Aluminum-rich silica slag, Zibo, China, Zongxiao Technology New Material Co.;
- Aluminum sulfate, analytically pure, Beijing, China, Beijing Kono Chemical Co., Ltd.;
- Aluminum fluoride, sodium sulfate, and aluminum hydroxide, analytically pure, Shanghai, China, Shanghai McLean Biochemical Technology Co.;
- Silicon Carbide, Anhydrous Ethanol and Deionized Water, St. Louis, MO, USA, Sigma-Aldrich Co.

The chemical composition of the aluminum-rich silica slag is shown in Table 1.

### 2.2. Laboratory Instruments

The test apparatus and experimental equipment used in this study were as follows:

Electronic Analytical Balance, accuracy 0.1 mg, Tianjin, China, Tianjin Deante Sensing Technology Co., Ltd.;

Vacuum-drying oven, Shanghai, China, Shanghai Shanzhi Instruments & Equipment Co., Ltd.;

X-ray diffractometer, Waltham, MA, USA, Thermo Fisher Scientific;

Scanning Electron Microscope, Waltham, MA, USA, Thermo Fisher Scientific;

Thermogravimetric analyzer, Zurich, Switzerland, METTLER TOLEDO GROUP, Switzerland;

Tensile strength tester, Shanghai, China, Stroma Industrial Co., Ltd.;

Muffle furnace, Shanghai, China, Li-Chen Instrument Technology Co., Ltd.

Planetary ball mill, Tianjin, China, Oriental Tianjing Technology Development Co., Ltd.;

Ceramic tablet press, Dongguan, China, Dongguan Kerui Instrument Co., Ltd.

### 2.3. Sample Preparation

#### 2.3.1. Mullite Whisker Preparation

**Figure 1:**
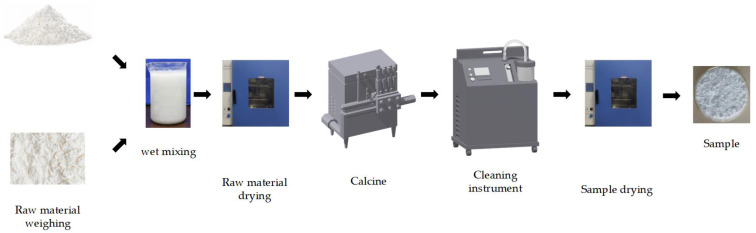
Mullite whisker preparation process.

In this study, silica-rich and aluminum-containing slag was used as the main silicon and aluminum source; aluminum sulfate and aluminum hydroxide were used as the supplemental source of aluminum ions, as well as the molten salt reaction system; and calcium fluoride and aluminum fluoride were used as the catalysts in the molten salt reaction system. The specific preparation process is shown in Figure 1. According to the proportions in Table 2, the raw materials were added to the mechanical stirring ball mill with a corundum ball. Deionized water was included with a mass ratio of 3:3:1. The rate of revolutions was 1200 r/min. We subjected the system to mechanical stirring for 12 h to ensure a fully mixed and homogeneous reaction of the slurry, which was then put into a vacuum drying oven at 120 °C for 10 h. Mantle milling was undertaken for 2 h until a fine powder was derived. This was then put in a corundum crucible to a 1/3 volume and placed in the muffle furnace, which was initially at room temperature. At 5 °C/min, the temperature was increased to 850 °C, and the heat was held for 5 h. We then removed the calcined samples and used deionized water to remove impurities (soluble salts). We repeated the rinsing, and the final samples were obtained by means of vacuum-drying to produce mullite whiskers.

#### 2.3.2. Whisker-Toughened Ceramic Composite Preparation

In this study, silicon carbide was used as the raw material for ceramic preparation, and mullite whiskers made in the laboratory were used as the toughening base for ceramics. In total, 5% of the raw material mass of the mullite whiskers and the ceramic preparation raw materials were put into a mechanically stirred ball mill, with the proportions of corundum spheres/total raw materials/deionized water at 3:2:1. With a rotational rate of 1200 r/min, mechanical stirring was carried out for 12 h to obtain a fully mixed and homogeneous reaction slurry. The homogeneous raw material slurry was put into the vacuum-drying oven at 100 °C to dry for 8 h; using a mortar and pestle, we ground the product for 2 h to a fine powder. This was then inserted into the ceramic tablet press machine for tablet molding, which was then put into the muffle furnace, initially at room temperature; the temperature was increased at a rate of 10 °C/min to 2000 °C, and insulation calcination was carried out for 3 h (heating program: first, set the furnace chamber temperature to the ambient temperature, then set a heating increase rate of 10 °C/min, and finally set the target temperature of 2000 °C, with a holding time of 3 h). We then removed the calcined samples and used anhydrous ethanol and deionized water for cleaning to remove impurities (soluble salts), rinsing repeatedly. The samples were then vacuum-dried.

### 2.4. Preparation Mechanism Analysis

#### 2.4.1. Mullite Whisker Growth Mechanism

The growth of mullite whiskers in the molten salt system was realized in a temperature gradient environment. A hot region and a cool region were established. In the hot region, the temperature was higher, while in the cooler region, the temperature was lower. This temperature gradient is critical because it causes mullite whiskers to precipitate out of the melt and grow.

As a result of the temperature gradient, supersaturated mullite material began to precipitate in the melt and form small crystal nuclei. These small nuclei gradually expanded and grew along the temperature gradient from the hot region to the cooler region. The presence of aluminum fluoride acted as a catalyst in this process and promoted whisker formation and growth. The fluoride ions interacted with the elements silicon and aluminum, lowering the energy barrier for growth and thus making whisker growth more likely.

Once whisker growth is complete, they were typically deposited on a suitable substrate, such as on the inside of a corundum crucible or another suitable support material. The growth rate, morphology, and orientation of the whiskers can be controlled by adjusting the temperature, the composition of the atmosphere, and the amount of aluminum fluoride added to the melt to achieve a mullite whisker material with the desired properties and morphology.

#### 2.4.2. Whisker-Toughened Ceramic Composite Material Mechanism

The mechanism for toughening ceramics with mullite whiskers involves a number of key factors, the interaction of which results in the superior toughness and strength of the ceramic material. (1) Crack arrest and dispersion: The hardness and strength of mullite whiskers are much higher than those of typical ceramic substrates, and when external stresses are applied to the ceramics, cracks often propagate from defects or stress concentration points. However, the presence of mullite whiskers prevents the uncontrolled expansion of cracks. The cracks are constrained around the whiskers and forced to change direction, which slows down the propagation of the cracks and thus improves the toughness of the material. (2) Energy absorption: When an external stress causes a mullite whisker to fracture, the whisker absorbs the strain energy. This energy is dispersed into the ceramic matrix, preventing rapid crack propagation. This energy-absorbing mechanism means crack expansion requires greater external stresses, and this improves the toughness of the material. (3) Interfacial effects: The interface between the mullite whiskers and the ceramic matrix also plays a key role. The properties of the interface, including interfacial bonding and compatibility, have an important influence on the material properties. By improving the interfacial bonding, the bonding between the whiskers and the substrate can be enhanced, stress concentration can be reduced, and the overall performance of the material can be further improved. (4) Size and distribution of whiskers: The size and distribution of whiskers also has an important impact on the performance of toughened ceramics. Usually, smaller and more uniformly distributed whiskers can provide better toughness enhancement because they more easily disperse stress, absorb energy, and prevent crack extension. Therefore, the size and distribution of whiskers need to be carefully controlled during the preparation process.

## 3. Results and Discussion

### 3.1. Effect of Different Molten Salt Systems

**Figure 2:**
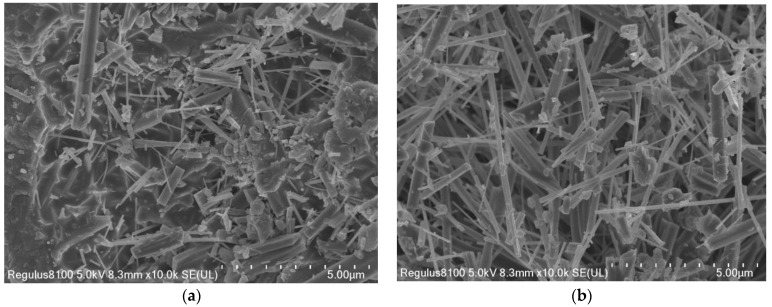
SEM images of mullite whiskers in different molten salt systems. (**a**) Aluminum hydroxide. (**b**) Aluminum sulfate.

In this subsection, aluminum hydroxide and aluminum sulfate are used as the supplemental aluminum source and the molten salt reaction system, respectively, in order to achieve the optimal whisker growth environment. SEM images of mullite whiskers composed from the aluminum sulfate and aluminum hydroxide molten salt reaction systems at a reaction temperature of 850 °C are shown in Figure 2. As shown in the figure, the aluminum hydroxide molten salt reaction system has more corundum or calcite phases; its whisker lengths and diameters are not uniform, and the whiskers are more fractured. However, in contrast, the mullite whiskers prepared from the aluminum sulfate molten salt system have fewer impurities, and the crystals are long and robust. The main reason for the above is that the mullite whiskers are affected by the concentration of aluminum ions and the reaction temperature. The melting temperature of aluminum hydroxide is much lower than that of aluminum sulfate, and much lower than the growth temperature of mullite whiskers, such that the aluminum hydroxide is completely molten at low temperatures, and the concentration of aluminum ions is saturated; but, at this time, the growth temperature of mullite whiskers has not been reached, meaning a corundum or calcite phase will be formed. On the contrary, the melting temperature of aluminum sulfate is close to the mullite whisker growth temperature, so when the aluminum sulfate is completely melted and the concentration of aluminum ions is saturated, the molten salt reaction occurs at the appropriate temperature for mullite whisker growth. In conclusion, the aluminum sulfate molten salt reaction system is the best system for mullite whisker preparation.

### 3.2. Effects of Different Reaction Temperatures

**Figure 3:**
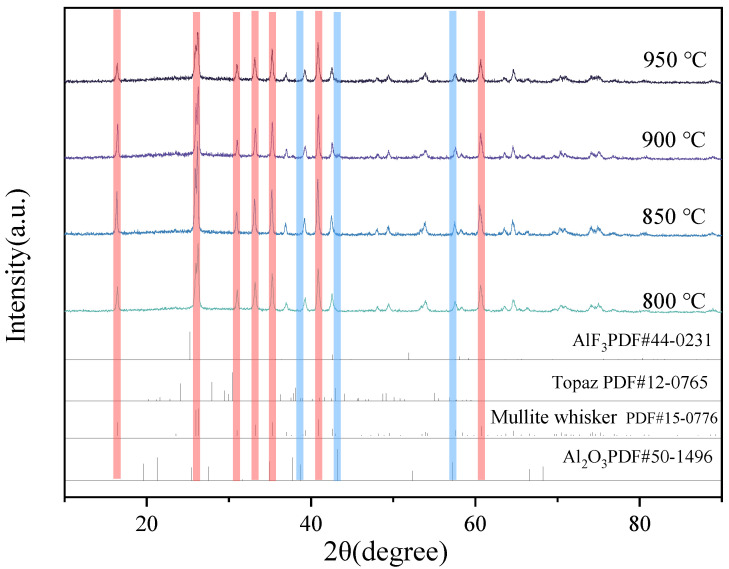
XRD of mullite whiskers prepared at different reaction temperatures (Red: mullite whiskers; blue: Al_2_O_3_).

XRD images of mullite whiskers prepared at reaction temperatures of 800 °C, 850 °C, 900 °C, and 950 °C with the addition of aluminum fluoride catalysts with mass fractions of 4% are shown in Figure 3. As shown, the phase compositions of the samples all have a whisker phase, i.e., a mullite crystalline phase, and all the schemes show strong diffraction peaks of the mullite crystalline phase at 25°. Comparing the aluminum fluoride and corundum phases, it was found that no stray peaks of aluminum fluoride appeared in the schemes with the same amount of aluminum fluoride addition, but stray peaks of corundum phases appeared at lower temperatures, and the presence of characteristic peaks can be observed at incident angles of 38° and 57°. The main reason for this is that the presence of aluminum fluoride during the growth of mullite whiskers acts as a catalyst for fluxing, and it can be melted at lower temperatures. When the aluminum fluoride is mixed with the raw material of mullite and heated at a high temperature, the aluminum fluoride starts to melt and forms a liquid phase. This liquid phase reduces the temperature at which the whiskers are prepared because diffusion between solid-phase particles occurs more readily in the presence of the liquid phase, which promotes whisker growth. Aluminum fluoride also reacts chemically with one of the main components in mullite, alumina (Al_2_O_3_), to form a fusible intermediate that helps to lower the sintering temperature. The main chemical equation for this reaction is given below:

AlF_3_·3H_2_O→AlF_3_ (s) + 3H_2_O(v)

AlF_3_(s)→AlF_3_ (v)

2AlF_3_ (v) + 3H_2_O(v)→Al_2_O_3_(s) + 6HF(v)

Al_2_O_3_ + 2HF→2AlOF(v) + H_2_O(v)

SiO_2_ + 4HF→SiF_4_ + 2H_2_O(v)

6AlOF + 2SiF_4_ + 7H_2_O→3Al_2_O_3_·2SiO_2_ (mullite whisker) + 14HF(v)

In a low-temperature molten salt system, aluminum fluoride reacts with water vapor to form gaseous AlOF and HF, which in turn react with silica to form SiF_4_ gas. When the concentration of AlOF and SiF_4_ is oversaturated, a high proportion of SiF_4_ and AlOF gas molecules precipitate at the interface of the molten salt, which leads to the chemical reaction between the molten salt and the steam to form mullite whiskers and HF, thus achieving the purpose of low-temperature catalyzed mullite whisker growth in the molten salt system.

**Figure 4:**
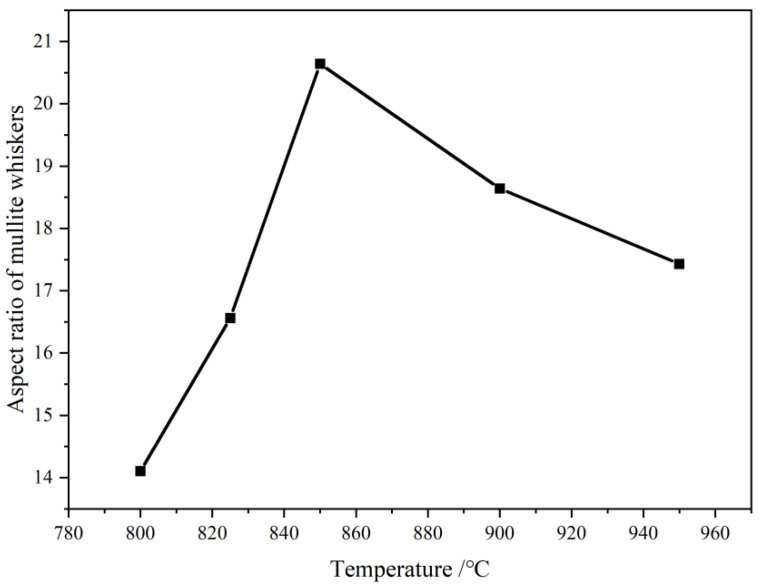
Mullite whisker aspect ratios at different reaction temperatures.

Comparing different molten salt reaction temperatures, it was found that the diffraction peak intensity of mullite whiskers showed a trend of enhancing and then weakening with the increase in sintering temperature. The diffraction peak intensity of the mullite whisker phase in the sample reaches its strongest value at the molten salt system temperature of 850 °C, at which the crystal structure is more complete—the higher the degree of ordering of the crystals, the better the degree of crystallinity, and the higher the purity of the mullite whiskers in the sample. When the preparation temperature is lower than 850 °C, the formation of the molten salt system will be incomplete, the temperature gradient will be small, and the growth of whiskers will be restricted. At too low a temperature, the ordering of the crystal lattice may be restricted, meaning it will be more difficult for the atoms or ions to move to the correct positions at low temperatures in order to form a fully ordered crystal structure. Similarly, when the temperature is higher than 850 °C, the following problems occur: (1) Crystal defects—At high temperatures, atoms or ions within the crystal may have more thermal vibrations, which help to reduce the ordering of the crystal. Defects and dislocations within the crystal may increase, resulting in an incomplete crystal structure. These crystal defects can scatter X-rays and diminish the intensity of the diffraction peaks. (2) Crystal growth and structural relaxation—At high temperatures, crystals of mullite whiskers may experience growth and structural relaxation. This may lead to small changes in the crystal structure, which may affect the XRD diffraction pattern. The relaxation of the crystal structure may lead to small changes in the lattice constants, which can also lead to changes in the position and intensity of the diffraction peaks. (3) Thermal rise and fall—At high temperatures, the thermal rise and fall (thermal vibration) of atoms or ions increases, which can lead to small irregularities in the crystal structure. These irregularities can be manifested in XRD as the blurring of diffraction peaks or a reduction in the intensity of the diffraction peaks. (4) Crystal particle size distribution—An increase in temperature may lead to a wider range of crystal particle sizes, i.e., an increase in the size variation of crystal particles. In XRD analysis, the increase in the size distribution of crystal particles may lead to the broadening of the diffraction peaks and a decrease in the intensity. Combined with Figure 4, the aspect ratio of the prepared mullite whiskers shows an increasing and then decreasing trend with the elevation of the preparation temperature, and the aspect ratio of the mullite whiskers reached the maximum of 20.64 when the preparation temperature was 850 °C. Therefore, the most suitable temperature for mullite whisker growth in this study was 850 °C.

**Figure 5:**
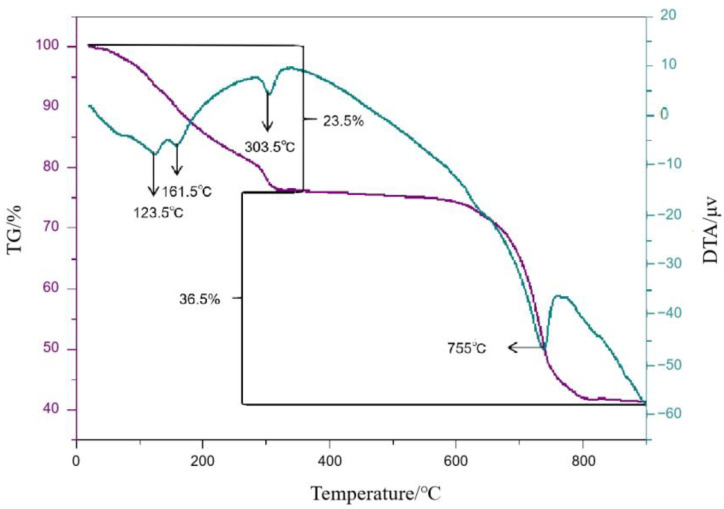
TG–DTA curves of samples of mullite whiskers in air from room temperature to 900 °C.

Figure 5 shows the TG curves of mullite whiskers, and the results show that the samples undergo obvious weight loss between 0 and 350 °C, accompanied by a 23.5% mass loss. The DTA curves show broad heat absorption peaks centered at 123.5 °C, 161.5 °C, and 303.5 °C, which can be attributed to the removal of crystalline water from the raw material of the reaction. The TG curves show that the second stage of weight loss begins to occur at 650 °C, which is due to the melting of aluminum sulfate. The total mass loss of 36.5% in the TG curve is mainly due to the partial decomposition of Al_2_(SO_4_)_3_. Since the decomposition of Al_2_(SO_4_)_3_ is a heat-absorbing process, the heat-absorbing peak appears at 755 °C in the corresponding DTA curve. No further weight loss was observed above 850 °C, indicating the complete decomposition of aluminum sulfate, where sodium sulfate did not decompose. Therefore, the reaction was completed at 850 °C.

From the above analysis, it can be concluded that the temperature conditions required for the stable growth of mullite whiskers are between 755 °C and 900 °C. When the sintering temperature increased from 755 °C to 850 °C, mullite whisker nucleation occurred, along with the beginning of growth, secondary growth, and the anisotropic growth of the morphology. Here, we see that 850 °C is the best sintering temperature for mullite whiskers; when the initial temperature is >850 °C, the crystallinity and aspect ratio of mullite whiskers decreases with increasing temperature.

### 3.3. Effects of Different Catalyst Additions

**Figure 6:**
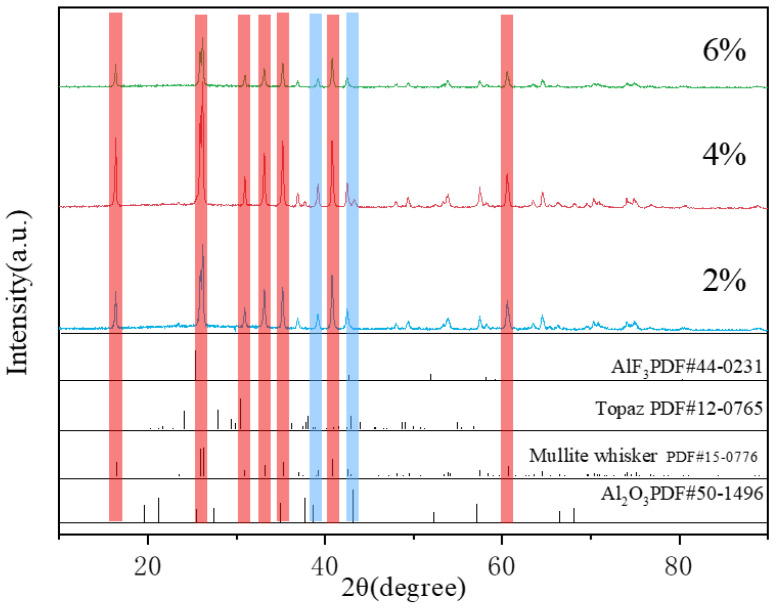
XRD of reaction systems with different mass fraction additions of aluminum fluoride (Red: mullite whiskers; blue: Al_2_O_3_).

Figure 6 shows XRD images of the reaction system with different mass fraction additions of aluminum fluoride in the molten salt reaction at a temperature of 850 °C. The results show that under the same temperature conditions of the molten salt reaction, with the increase in aluminum fluoride addition, we see by comparing the characteristic peaks of the standard mullite whiskers that the intensity of the XRD diffraction peaks of the samples at 39° and 42° tends to be stronger and then weaker. The maximum intensity of the diffraction peaks was reached at 4% of aluminum fluoride addition. The main reason for this phenomenon is that, at the initial stage, when aluminum fluoride is added to the mullite, it may cause a slight adjustment of the crystal structure, which can lead to an increase in the intensity of the XRD diffraction peaks. This enhancement may be due to the fact that some of the chemical properties of aluminum fluoride are compatible with the crystal structure of mullite, thus inducing a more ordered arrangement of the crystal lattice, which increases the intensity of the diffraction signal. With further increases in the addition of aluminum fluoride, a saturation point may be reached, or a suitable amount of addition may be exceeded. At this point, the aluminum fluoride may begin to occupy too many lattice positions, causing the crystal structure to become disordered or unstable. This instability can lead to partial disintegration of the crystal structure, which weakens the intensity of the XRD diffraction peaks. The excessive addition of aluminum fluoride may also introduce crystal defects, such as point defects, dislocations, or heterogeneous interfaces, which can scatter X-rays, resulting in a reduction in the intensity of the diffraction peaks. Excessive amounts of aluminum fluoride may initiate phase transitions in mullite, leading to changes in the crystal structure, which may result in changes in the position and intensity of the XRD peaks.

**Figure 7:**
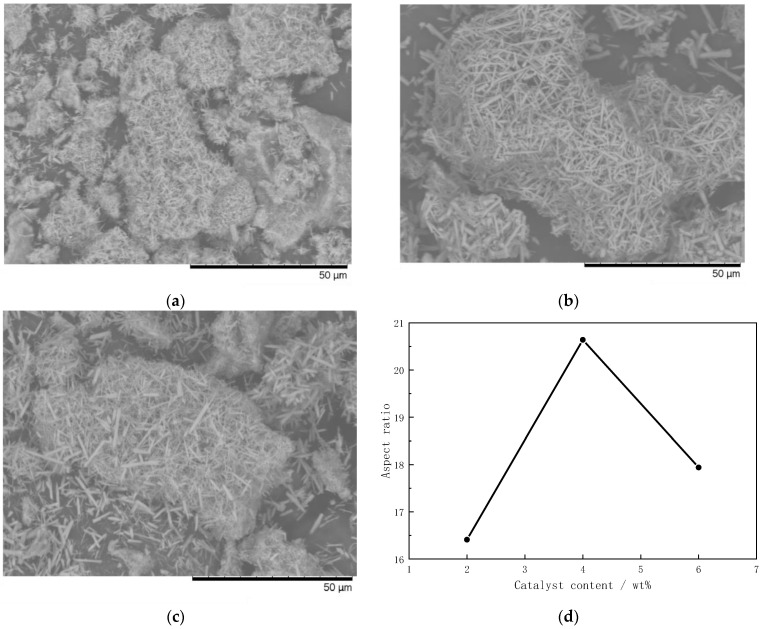
SEM images and trends in aspect ratio of mullite whiskers prepared with different catalyst additions. (**a**) Two percent. (**b**) Four percent (**c**) Six percent. (**d**) Trends in aspect ratio.

Figure 7 illustrates the trends within the SEM and aspect ratio findings for mullite whiskers prepared with different additions of aluminum fluoride catalyst. When the additional amount of aluminum fluoride catalyst was 2%, there were obvious clusters at the bottom and top of the whiskers, and the whiskers were small and short, with an average aspect ratio of 16.41. When the additional amount of aluminum fluoride catalyst was 4%, the whiskers were stout and long, with no obvious clustering phenomena, and the dispersion was uniform, with an average aspect ratio of 20.64. When the additional amount of aluminum fluoride catalyst was 6%, the phenomenon of top agglomeration also appeared, the dispersion was not uniform, the whiskers were shorter, and the average aspect ratio was 17.94. The results show that the additional amount of aluminum fluoride positively affected the crystallization of mullite whiskers and the aspect ratio, but the additional amount should not be too large—the optimal additional amount was 4%.

### 3.4. Effect of Heating Holding Time

**Figure 8:**
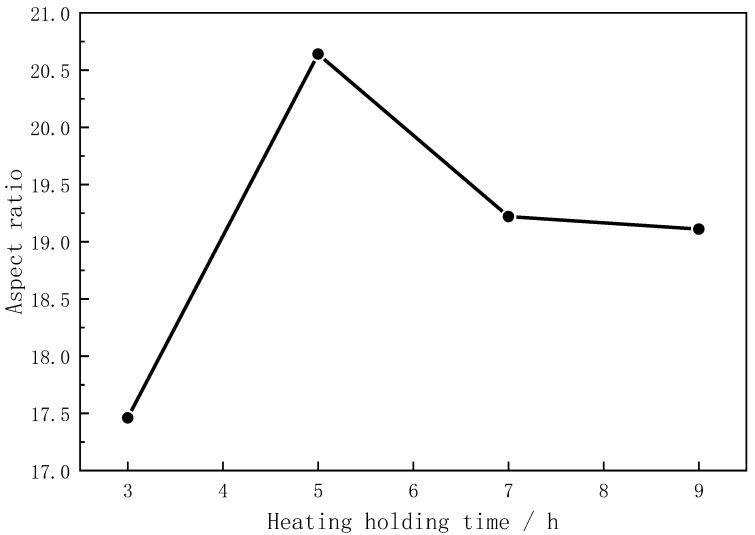
Aspect ratio of mullite whiskers prepared with different heating holding times.

**Figure 9:**
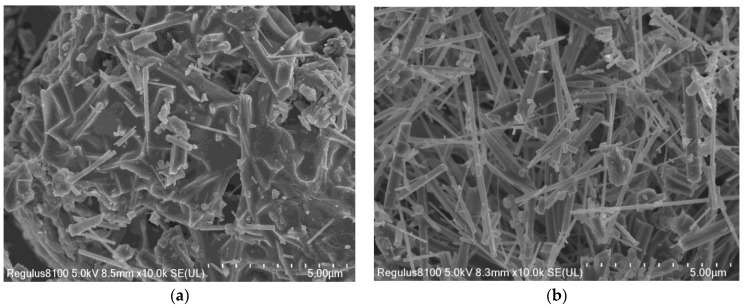
SEM images of mullite whiskers with different heating holding times. (**a**) Three hours. (**b**) Five hours.

Different holding times were adopted in the formulation with a 4% catalyst addition and an 850 °C reaction temperature to find the optimal mullite whisker preparation scheme. In Figure 8, the trend graphs of the variation in the aspect ratio of mullite whiskers prepared with different holding times are compared. The results show that, under the same preparation scheme, the aspect ratio of mullite whiskers increased and then decreased with the increase in the holding time, and reached the highest value at 5 h. Combined with the SEM images in Figure 9, it can be seen that the mullite whiskers prepared with a 5 h holding time had thicker crystal skeletons, more complete crystal phases, and larger aspect ratios compared to the 3 h holding time. The main reason for this phenomenon is that an insufficient holding time leads to the incomplete growth of whiskers, while excessive holding times lead to whisker fracture and, thus, reductions in the aspect ratio.

### 3.5. Effect of Temperature Rise Rate

**Figure 10:**
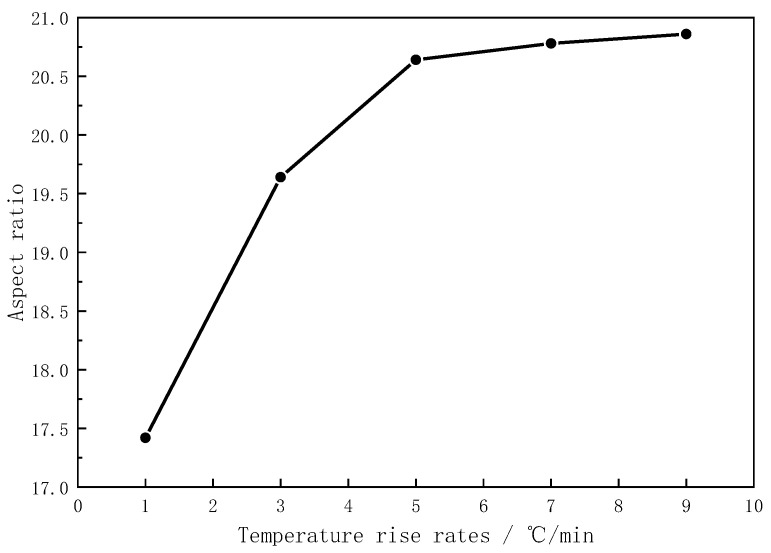
Aspect ratio of mullite whiskers prepared with different temperature rise rates.

**Figure 11:**
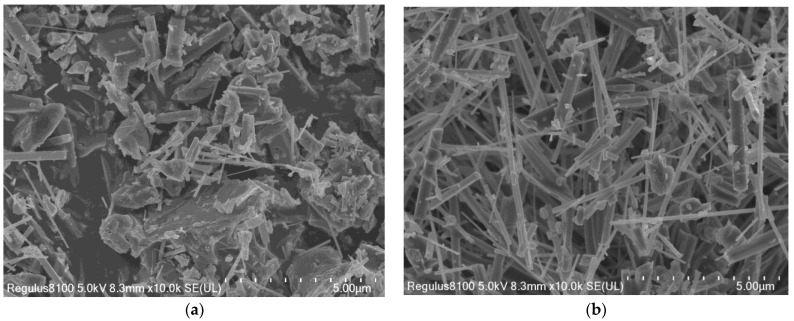
SEM images of mullite whiskers with different temperature rise rates: (**a**) 1 °C/min; (**b**) 5 °C/min.

Different temperature rise rates were adopted in the formulation with a 4% catalyst addition and an 850 °C reaction temperature to find the optimal mullite whisker preparation scheme. In Figure 10, the trend plots of the variations in the aspect ratios of mullite whiskers prepared at different temperature rise rates are compared. The results show that the aspect ratios of mullite whiskers increased with the increase in the heating rate under the same preparation scheme, but the change in the whisker aspect ratio was not obvious when the heating rate was greater than 5 °C/min. For example, the aspect ratio of mullite whiskers increased by 18.48% when the temperature increase rate was increased from 1 °C/min to 5 °C/min, but the aspect ratio of mullite whiskers increased by 1.07% when the temperature increase rate was increased from 5 °C/min to 10 °C/min. Meanwhile, in combination with the SEM images shown in Figure 11, it was found that the mullite whiskers prepared under the 5 °C/min scheme were more uniformly dispersed, structurally more complete, and had a larger aspect ratio than those prepared under the 1 °C/min scheme. Therefore, in order to control the preparation cost of mullite whiskers, it is better to use the 5 °C/min scheme.

### 3.6. Effects of Different Additives on Toughening Properties of Ceramics

**Figure 12:**
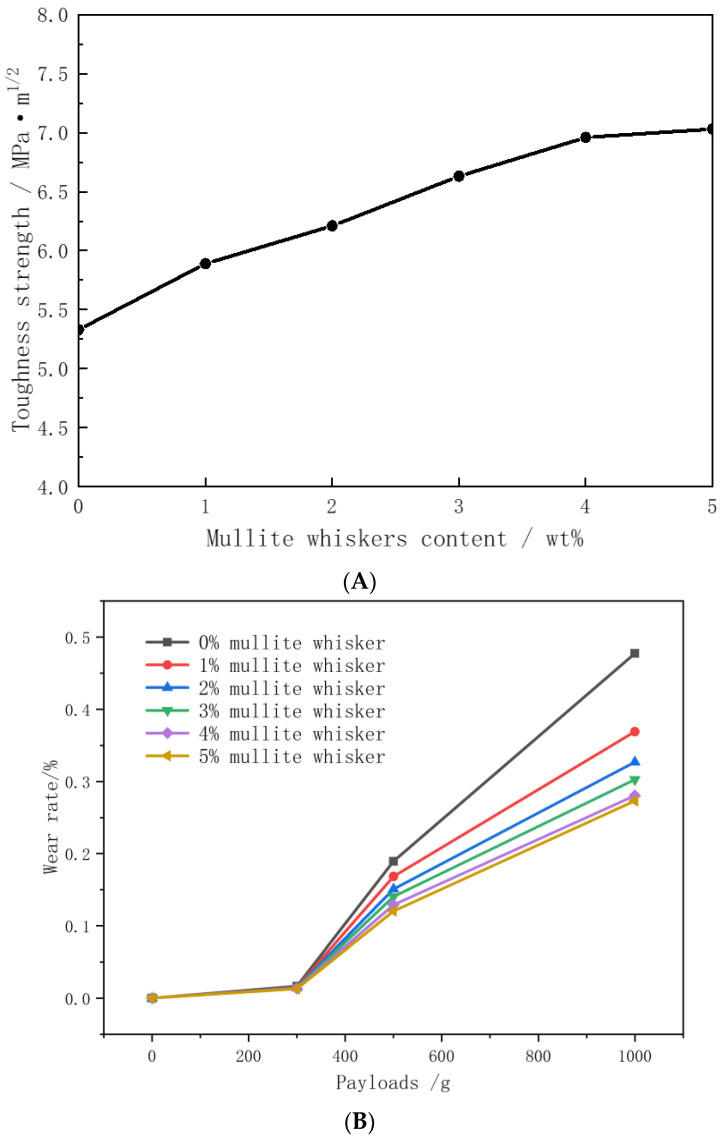

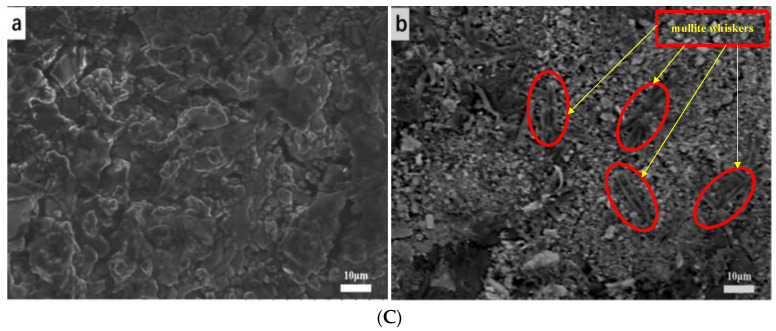
The SEM images and trend graphs of toughness strength and wear rate of ceramics with different mullite whisker additions. (**A**) Toughness strength. (**B**) Wear rate. (**C**) SEM images of 0% (**a**) and 3% (**b**) mullite whiskers.

Mullite whiskers were added to ceramics at 1%, 2%, 3%, 4%, and 5%, and the strengths of the toughened ceramics were tested using a universal testing machine. As shown in Figure 12, the trend graphs of the toughness strength and wear rate of ceramics with different mullite whisker additions were compared. The results show that the toughness strength and wear rate of ceramics increased with the increase in mullite whisker addition. However, with the increase in mullite whisker addition, the toughening amplitude of the toughness strength and wear rate of ceramics gradually decreased, especially when the mullite whisker addition was more than 3 wt. %, and the continued increase in mullite whisker content led to a limited enhancement in the wear resistance of ceramics. For example, at a load of 1000 g, the wear rate of the ceramic was reduced by 36.61% when the mullite whisker addition was increased from 0% to 3%, but the wear rate of the ceramic was reduced by 9.75% when the mullite whisker addition was increased from 3% to 5%. Combining the SEM images with mullite additions of 0 wt. % and 3 wt. %, it can be seen that with the addition of mullite whiskers, cracks often started to expand from defects or stress concentration points when external stresses were applied to the ceramics. However, the presence of mullite whiskers prevented the uncontrolled extension of cracks. The cracks were restrained around the whiskers and forced to change direction, which slowed down crack propagation and, thus, increased the toughness of the material. Considering the economic costs, the addition of 3 wt. % of mullite whiskers as a toughening base enhanced the toughness strength of the ceramics by 16.56% and reduced the wear rate to less than 0.4% compared to the addition of no mullite whiskers.

## 4. Conclusions

In this paper, the effects of using silica-rich slag with different molten salt systems, catalyst additions, preparation temperatures, heating holding times, temperature rise rates, and drying modes on the preparation of mullite whiskers were investigated, and the toughening effect of mullite whiskers on silicon carbide ceramics was also further investigated. The following conclusions were drawn:

1. Compared to the molten salt system of aluminum hydroxide, the molten salt system of aluminum-sulfate-prepared mullite whiskers showed a purer crystal phase, no excess impurity phase, and a large aspect ratio.
2. The effects of different reaction temperatures, catalyst additions, heating holding times, and temperature rise rates in the same molten salt system were considered. Among these, the effects of different reaction temperatures, catalyst additions, and heating holding times on the aspect ratio of mullite whiskers showed a tendency of increasing and then decreasing. The temperature rise rate had a positive effect on the growth of mullite whiskers, but the effect was not obvious when the temperature rise rate exceeded 5 °C/min.
3. To benefit economic costs, the aluminum sulfate molten salt system was selected, the aluminum fluoride catalyst addition was set at 4%, and the preparation temperature was raised to 850 °C at a temperature increase rate of 5 °C/min before being held for 5 h. The aspect ratio reached 20.64.
4. The toughening effect of mullite whiskers on ceramics was obvious—the optimal addition of mullite whiskers is suggested to be more than 3 wt. %. Compared with those of ceramics without mullite whiskers, the toughness strength was enhanced by more than 16.5%, and the wear rate was reduced by 36.61% to a value lower than 0.4%.

## Figures and Tables

**Table 1:** Composition of aluminum-rich silica slag.

Ingredients (wt. %)	Content Percentage
Al_2_O_3_	28.7
SiO_2_	49.4
Na_2_O	17.2
SO_3_	4.5
Other trace oxides	0.2

**Table 2:** Proportions of raw materials.

Proportions of Raw Materials	Mass Ratio
Aluminum-rich silica slag	10
Catalysts	1.7
Molten salt-based raw materials	5

## Data Availability

The data presented in this study are available upon request from the corresponding author. The data are not publicly available due to the laboratory interests involved in this data.
